# Pulmonary metastasectomy in colorectal cancer: A letter in response to Antonoff and colleagues

**DOI:** 10.1016/j.xjon.2021.08.031

**Published:** 2021-08-26

**Authors:** Fergus Macbeth, Joel Dunning, Tom Treasure

**Affiliations:** a Centre for Trials Research, Cardiff University, Cardiff, United Kingdom; b James Cook University Hospital, Middlesbrough, United Kingdom; c Clinical Operational Research Unit, University College, London, United Kingdom

To the Editor:

**Figure:**
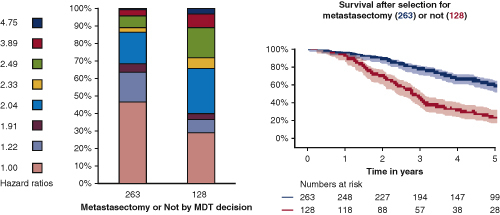

The authors reported no conflicts of interest.

The *Journal* policy requires editors and reviewers to disclose conflicts of interest and to decline handling or reviewing manuscripts for which they may have a conflict of interest. The editors and reviewers of this article have no conflicts of interest.

The review of pulmonary metastasectomy by Antonoff and colleagues^1^ suggests that the aim of treatment is local control with an implicit assumption that this leads to survival benefit. They state that “there is clear demonstration of improvement in prognosis for appropriately selected surgical patients,” “Surgery has also been shown to extend survival for patients with metastatic sarcoma,” and “Surgical therapy has further been found to be beneficial for pulmonary metastases from renal cell cancer as well as melanoma.”^1^ None of these statements is supported by controlled trial evidence. This is no more than strong but unsubstantiated belief.

The Pulmonary Metastasectomy in Colorectal Cancer (PulMiCC) randomized controlled trial (RCT) was run in the face of such widespread conviction. We did not claim that PulMiCC ruled out any survival benefit but pointed out that median survival was actually longer in the control group, at 3.8 years versus 3.5 years.^2^ Use of chemotherapy or local ablation was similar. If a Phase 2 evaluation of a novel cancer drug found a modest survival difference in favor of the control arm it would probably lead to a halt in further investigation of the drug because the chance that it would be clinically useful or commercially viable would be too remote. Why should such considerations not apply to metastasectomy?

**Figure 1:**
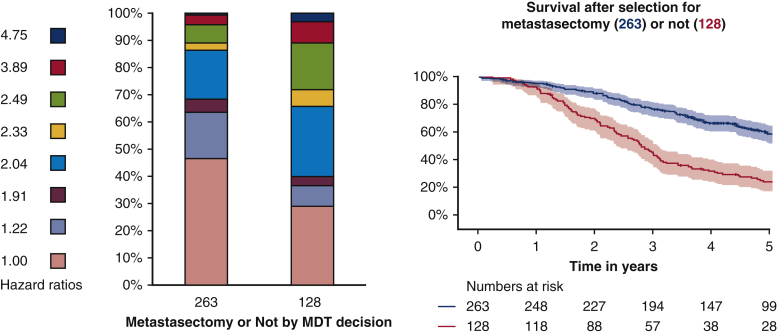
In the bar charts on the left, the proportions of 263 patients who had metastasectomy and 128 who did not by cancer team decisions^3^ are color coded according to the hazard ratios. As calculated in a meta-analysis of 2925 patients, these were 2.04, 1.91, and 1.22 for multiple metastases, elevated carcinoembryonic antigen, and treated liver metastases, respectively.^4^ For patients who had 2 or 3 of these hazards the products of the hazard ratios have been derived. On the right, the product-limit survival estimates with numbers at risk and 95% confidence intervals are presented. The difference is largely, and could be entirely, attributable to the differences in the known adverse factors.

PulMiCC included 512 patients. This is not “poor recruitment” as stated by Antonoff and colleagues.^1^ The proportion randomized was limited because of the beliefs exemplified above. We closed PulMiCC because it was important to analyze the data we had. Elective decisions were for metastasectomy in 263 and against in 128.^3^ Without metastasectomy, 5-year survival was 22% (95% confidence interval, 15%-30%) (see Figure 1), significantly better than the implausible “worse than 5%” assumption (*P* < .001).^4^ Falsely low estimates create an impression of benefit from treatment.

Qi and Fan's^5^ statement, “If patients do not receive timely and effective treatment, they may die as a result of respiratory failure” is also incorrect. Lung metastases rarely contribute substantially to death or terminal symptoms. These patients might be better served by receiving this reassuring information and being spared pointless surgery. Lung metastases are the most easily imaged component of what is nearly always systemic disease and they can be monitored for progression and response to proven systemic treatments.

Baseline data were collected on all PulMiCC patients to RCT standard. Contrary to the incorrect statement, “The majority of patients enrolled [in the RCT] displayed highly favorable characteristics,”^1^ the randomized patients had a mix of risk factors somewhere between the elective groups. The bar graph illustrates how differently these hazards were represented. The survival between the 2 groups is less than half that widely claimed and could all be attributable to the difference in the known adverse factors.^4^

It took 90 years before RCTs showed that women with breast cancer could be spared mutilating radical surgery. There is no trustworthy evidence that a policy of pulmonary metastasectomy results in more than a few anecdotal cures and Gray and Molena^6^ must know that to cut is a chance to harm.
